# The footprint of human-induced climate change on heat-related deaths in the summer of 2022 in Switzerland

**DOI:** 10.1088/1748-9326/ace0d0

**Published:** 2023-07-04

**Authors:** Ana M Vicedo-Cabrera, Evan de Schrijver, Dominik L Schumacher, Martina S Ragettli, Erich M Fischer, Sonia I Seneviratne

**Affiliations:** 1 Institute of Social and Preventive Medicine, University of Bern, Bern, Switzerland; 2 Oeschger Center for Climate Change Research, University of Bern, Bern, Switzerland; 3 Graduate School of Health Sciences, University of Bern, Bern, Switzerland; 4 Institute for Atmospheric and Climate Science, ETH Zürich, Zürich, Switzerland; 5 Swiss Tropical and Public Health Institute (SwissTPH), Allschwil, Switzerland; 6 University of Basel, Basel, Switzerland

**Keywords:** mortality, climate change, heat, attribution

## Abstract

Human-induced climate change is leading to an increase in the intensity and frequency of extreme weather events, which are severely affecting the health of the population. The exceptional heat during the summer of 2022 in Europe is an example, with record-breaking temperatures only below the infamous 2003 summer. High ambient temperatures are associated with many health outcomes, including premature mortality. However, there is limited quantitative evidence on the contribution of anthropogenic activities to the substantial heat-related mortality observed in recent times. Here we combined methods in climate epidemiology and attribution to quantify the heat-related mortality burden attributed to human-induced climate change in Switzerland during the summer of 2022. We first estimated heat-mortality association in each canton and age/sex population between 1990 and 2017 in a two-stage time-series analysis. We then calculated the mortality attributed to heat in the summer of 2022 using observed mortality, and compared it with the hypothetical heat-related burden that would have occurred in absence of human-induced climate change. This counterfactual scenario was derived by regressing the Swiss average temperature against global mean temperature in both observations and CMIP6 models. We estimate 623 deaths [95% empirical confidence interval (95% eCI): 151–1068] due to heat between June and August 2022, corresponding to 3.5% of all-cause mortality. More importantly, we find that 60% of this burden (370 deaths [95% eCI: 133–644]) could have been avoided in absence of human-induced climate change. Older women were affected the most, as well as populations in western and southern Switzerland and more urbanized areas. Our findings demonstrate that human-induced climate change was a relevant driver of the exceptional excess health burden observed in the 2022 summer in Switzerland.

## 1 Introduction

During the last few years, we have been witnessing how extreme weather events are becoming the norm in our daily lives. In particular, 2022 can be considered a year of records concerning weather and climate. The severe floods in Pakistan and some parts of Western Africa, the extreme drought affecting parts of the Northern Hemisphere and the extremely warm summer in Europe and China are all clear manifestations of this worrying trend [1]. Climate change has strongly changed the odds of these extreme events with some events being implausible in the absence of human-made warming [2–6]. More importantly, these extreme events are responsible for a substantial health burden, of which a large proportion can be directly attributed to anthropogenic climate change [7–9]. In particular, heat is an important driver of health burden responsible for nearly 1% of annual all-cause deaths worldwide [10]. Moreover, a recent investigation found that around 37% of heat-related mortality since the 1990s can be attributed to human-induced climate change [8]. However, there is limited quantitative evidence on the footprint of anthropogenic climate change on the health burden of recent extreme weather events.

The European summer of 2022 was characterized by a cascade of heatwaves, especially in West–Central Europe. Many regions in Europe experienced the strongest anticyclonic conditions since at least 1950, fostering descending and warming air masses, inhibiting convection and leading to cloud-free conditions that in turn enabled unusually high insolation [11]. At the same time, precipitation was below average across most of Europe, so the warm and dry weather conditions also manifested in widespread soil desiccation, which likely further amplified the extremely high air temperatures [4, 12, 13]. As such, all ingredients for an exceptionally hot summer were present. But the summer of 2022 was also particularly devastating in terms of exceptionally high health burden, with twice and even four times larger than the excess mortality burden in the same period of 2021 and 2020, respectively [14]. The still ongoing COVID-19 pandemic and the exceptionally hot and dry weather in Europe—and other related environmental hazards (e.g. wildfires, poor air quality)—might have created a *perfect storm* resulting in a substantial mortality burden in many European countries. These hazards, in particular, heat and COVID-19 mostly affect the same vulnerable population (i.e. older adults and other frail individuals) [15–17]. However, the specific contribution of each of these drivers to the observed excess burden remains unclear.

Under current rates of warming, the summer of 2022 becomes an average season already in the coming decades [18]. Additionally, in absence of efficient adaptation strategies, the progressive ageing of the population and the (re-)emergence of infectious diseases would potentially lead to even larger health impacts. Thus, quantitative evidence on the health impact attributed to anthropogenic activities during such exceptional summer could be tremendously impactful demonstrating that climate change is an unfolding environmental crisis which is already exacerbating the health burden of climate-sensitive diseases. This evidence would help increase awareness and boost action, inform the development of adaptation and mitigation strategies, and provide valuable evidence in climate litigation [19, 20]. Thus, we here aimed to quantify the contribution of human-induced climate change to the observed mortality due to heat in Switzerland during the summer of 2022. As for most of western and central Europe, summer 2022 was exceptionally hot and dry in Switzerland. Average temperatures between June and August 2022 ranked second since the beginning of the records, exceeded only by the infamous summer of 2003 when a large increase in excess mortality was reported in this country [21, 22]. Additionally, according to the official federal estimates, this season was exceptionally severe in terms of excess mortality with nearly 1800 excess deaths among the population aged 65 and older between June and August 2022 [23].

## 2 Methods

We applied state-of-the-art methods in climate epidemiology and attribution science in combination with high-resolution temperature-mortality data to derive robust estimates of the contribution of human-induced climate change.

### 2.1 Estimation of the temperature-mortality associations

To quantify the mortality burden associated with heat, we first need to assess how the mortality risk changes with temperature across subpopulations in Switzerland. In brief, for each sex-age (0–65 and above 65 years) subgroup, we derived the temperature-mortality association (pooled or nation-wide, and canton-specific risks) through a two-stage time-series [24] analysis using high-resolution temperature and mortality data.

We collected mortality data in Switzerland between 1990 and 2017 from the Federal Office of Statistics (supplementary table 1). Mortality data after 2017 at the municipality level was not available at the time of the analysis. We derived daily all-cause counts by combinations of sex and age groups in each municipality, as described in a recent publication [25]. For the same study period, we derived daily population-weighted temperature series in each municipality by combining a high-resolution 2 km-cell temperature data (MeteoSwiss) [26], with gridded population data in Switzerland (2010 EOSDIS gridded population, 1 km) (supplementary table 1) [27]. Previous investigations showed that population-weighted metrics would better account for the heterogeneous distribution of the population due to the irregular topography in Switzerland [28]. Since we were interested in heat, we restricted the analysis to the summer months between June and August.

We performed a two-stage time-series analysis to derive the canton-age-sex-specific exposure-response associations, following a similar procedure of a recent publication [25]. This is the standard methodology used in multi-location temperature-mortality assessments [29, 30]. In particular, we first performed case time-series analysis to estimate the canton-specific temperature-mortality association. This novel methodology is being extensively applied in the assessment of short-term associations in small-area units [31, 32]. The method allows using the exposure and outcome disaggregated at small-area units (i.e. municipality), thus capturing differential changes in exposure across space and time, reducing the potential exposure misclassification and increasing the statistical power (supplementary methods 1). The temperature-mortality association was modelled using the distributed lag non-linear framework, that properly accounts for non-linear and delayed dependencies, in this case up to 10 days of lag [29].

In the second stage, for each combination of sex and age subgroups, we performed a random-effects multivariate multilevel meta-analysis of the canton-specific exposure-response associations estimated in the previous step [30] following the procedure of De Schrijver *et al* (2022) (supplementary methods 1). We report the heat-mortality association in terms of relative risks (RRs) and the corresponding 95% confidence intervals (CI) at each temperature value above the temperature of minimum mortality (MMT), used here as a reference. MMT can be considered the optimum temperature corresponding to the minimum mortality risk between the 25th and 90th percentile, consistently with previous assessments [24].

### 2.2 Estimation of the heat-related mortality in summer 2022

We collected daily all-cause mortality by sex and age category between June and August 2022 from the Federal Office of Statistics. Given the short period between the registration of the deaths and when this data was provided, these are the provisional number of death counts (i.e. late reported cases not considered, and corrections are not yet made) only available at the cantonal level. We also derived the population-weighted daily mean temperature series in each canton between June and August 2022 using the same data source and procedure as for the period 1990–2017.

We quantified the all-cause heat-related mortality using the observed daily mortality and the risk estimate of the exposure-response association corresponding to the observed mean temperature each day [33]. We applied this same procedure to each of the canton-sex-age series. Heat-related mortality burden corresponds to the sum of all deaths attributed to temperature in days with mean temperature above the MMT. Heat-mortality burden in each day accounts for the cumulative risk in the preceding 10 days [33]. We quantified the uncertainty of the impact estimates by computing 1000 samples of the set of coefficients defining the overall cumulative exposure-response association (i.e. accounting for the risks across 10 d of lag) derived through Monte Carlo simulations and assuming a multivariate normal distribution. We represented the level of uncertainty with the 95% empirical confidence intervals (95% eCI) derived as the 2.5th and 97.5th percentiles of the resulting distribution.

### 2.3 Health attribution analysis

Climate change attribution studies quantify the effect of anthropogenic climate change on the intensity or probability of an individual weather or climate event, or on a long-term trend in a climate variable relative to a counterfactual climate in which human influence is excluded [34]. In this study, we quantified the contribution of human-induced climate change as the difference between the observed heat-related mortality and the hypothetical burden assuming a counterfactual scenario of a climate in absence of an anthropogenic signal.

To compute the counterfactual temperature time series, we first estimated the mean human-induced warming level for June–August using daily mean temperatures averaged over Switzerland from both observations (gridded E-OBS data v25.e and an area-averaged temperature time series from MeteoSwiss [35–37]) and climate models. We also used simulated and observed (HadCRUT5 [38]) global mean surface temperature (GMST), and employed historical CMIP6 simulations extended in the last few years with the SSP5-8.5 scenario [39], extracting the summertime daily mean temperature over Switzerland and GMST for a total of 25 models, always using the first available ensemble member. The warming levels were estimated by linearly regressing average Swiss summertime temperatures on GMST, either combining observations (MeteoSwiss or E-OBS and HadCRUT5) or using only climate model output (i.e. we infer the (linear) relationship between mean June and August temperatures in Switzerland and global warming) (supplementary methods 2). This resulted in a warming level of 2.16 °C according to E-OBS, 2.75 °C with MeteoSwiss data, and a range of 1.19–2.27 °C based on the CMIP6 models, calculated as the intermodal mean *±* 1 standard deviation. Since the observed global warming since pre-industrial times is mainly anthropogenic (IPCC 2021), we derived four counterfactual temperature series between June and August 2022 by subtracting these four warming levels from the cantonal population-weighted daily mean temperatures.

Next, we computed the heat-related mortality attributable to human influence as the difference between the observed deaths due to heat (i.e. using the observed temperature) and the corresponding burden estimated with the four-counterfactual series between June and August 2022. The impacts in the latter were estimated using the same procedure as described above for the computation of heat-related mortality. We derived an ensemble estimate for the counterfactual scenario by computing the average impacts across the four series. The corresponding 95% eCI in this case was then estimated by combining the impact distributions derived from the simulated coefficients—thus, accounting for both the uncertainty of the risk estimates (i.e. exposure-response curve) and the variability across counterfactual series.

## 3 Results

### 3.1 Association between heat and all-cause mortality in Switzerland

**Figure 1:**
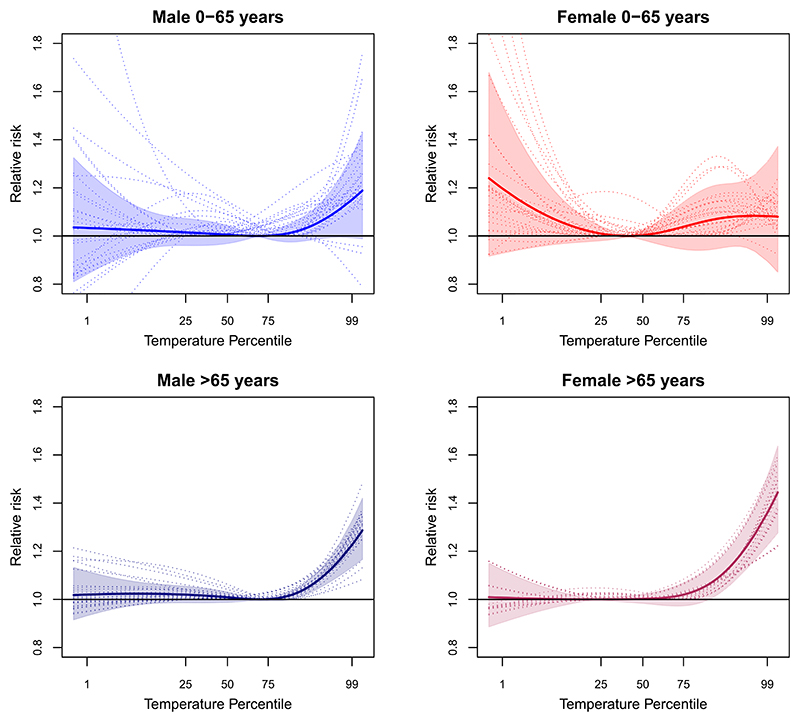
Age-sex-specific exposure-response curves summarizing the overall cumulative temperature-mortality risk in Switzerland (1990–2017) along 10 d of lag. Solid lines represent the pooled nationwide risks, expressed as relative risks, derived from the meta-analysis of the canton-specific exposure-response curves (dotted lines correspond to the BLUPs of the canton-specific associations). The shaded area corresponds to the 95% confidence interval of the pooled estimates. Risks are estimated as change in mortality risk at each temperature percentile of the nationwide temperature distribution versus the corresponding temperature of minimum mortality (MMT).

Figure 1 shows the pooled nationwide and canton-specific temperature-mortality associations by age-sex groups in Switzerland (detailed curves in supplementary figure 1, and heat-mortality RR in each age-sex-canton combination and nationwide (or pooled) in supplementary table 2). The pooled curves in figure 1 show the typical reversed ‘L’-shape of summer-only analysis with increased risks in the right tail of the curve from the MMT to the maximum temperature value. Curves are highly heterogeneous between sex-age subgroups and cantons, although overall we can observe a common pattern of larger vulnerability to heat in older adults (i.e. the steeper slope of the right tail of the curve). In particular, females aged above 65 years are on average more affected with a 36%-increase in mortality risk associated with heat (RR = 1.36 [95%CI: 1.22–1.51]), compared to 23% in the male counterparts (1.23 [95%CI: 1.13–1.33]). In contrast, males aged 65 and below in Switzerland show larger vulnerability compared to females of the same age (1.15 [95%CI: 0.99–1.33], vs. 1.08 [95%CI: 0.90–1.31]). Instead, younger females show increased mortality risk with decreasing temperatures at cooler temperature ranges suggesting an effect of cold-summer temperatures. Although estimates in this age group were highly uncertain and risks widely varied across cantons due to the low statistical power.

### 3.2 Heat in Switzerland during the summer of 2022

**Figure 2:**
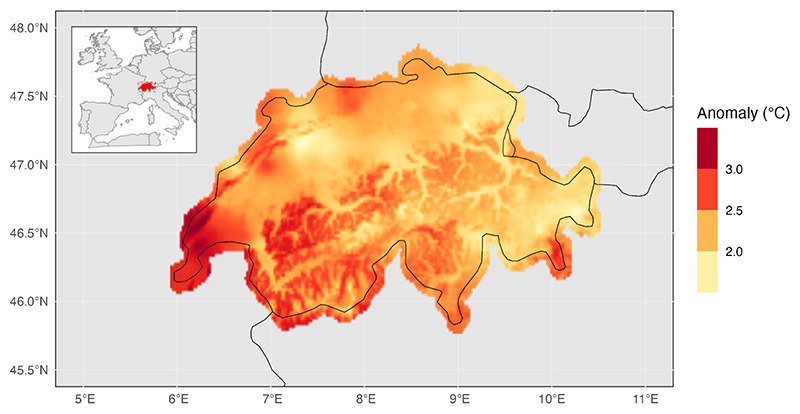
Map showing temperature anomaly in the summer of 2022 (June-August) (reference: summer 1990–2017). Temperature anomaly is estimated as the difference between the summer average temperature in 2022 vs. the 1990–2017 average in each 2 km cell across Switzerland.

On average, daily mean temperature was 2.3 °C higher than the 1990–2017 mean (range: 1.6 °C–3.3 °C). Southern and southwestern Switzerland (i.e. cantons of Geneva, Vaud and Ticino) were particularly affected with higher anomalies (figure 2). The highest daily mean temperatures (population-weighted) were recorded in the cantons of Basel-Stadt (28.7 °C) and Geneva (28.7 °C) (supplementary table 3).

### 3.3 Heat-related mortality during the 2022 summer in Switzerland

**Figure 3:**
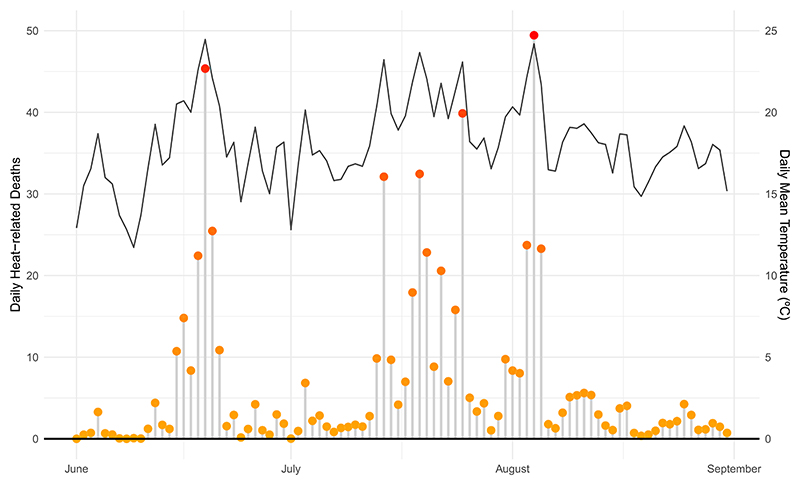
Daily heat-related deaths (orange dots) and daily mean temperature (black line) in Switzerland in summer 2022. The former corresponds to the sum of heat-related deaths across all age-sex subgroups and cantons estimated from the specific exposure-response curves (Methods). Mortality burden in each day accounts for the cumulative risk in the following 10 d. Daily mean temperature displayed here is the daily average of the population-weighted mean temperature series across the 26 cantons in Switzerland.

We used the sex-age-specific temperature-mortality risks in each canton to calculate the number of deaths associated with heat in the 2022 summer. Overall, we estimated that 623 [95%eCI: 151–1068] all-cause deaths can be attributed to heat between June and August 2022, representing 3.5% [95%eCI: 0.9–6.1] of total all-cause mortality during the same period. During the warmest days (>20 °C daily mean) more than 30 deaths can be attributed to heat each day (figure 3), corresponding to more than 15% of the total all-cause deaths per day (supplementary figure 2). Nearly 90% of the estimated heat-related mortality happened in older adults (544 [95%eCI: 161–914]) (supplementary table 4). However, deaths due to heat represented a similar proportion of the total all-cause deaths in both age groups with 3.5% [95%eCI: 1.1–5.9] in those above 65 years and 3.6% [95%eCI: *−*1.2 to 7.4] in the 0–65 years. Heat-related mortality in the 2022 summer was larger in women with 372 deaths (4.1% [95% CI: 0.7–7.3] of all-cause deaths vs. 251 deaths corresponding to 2.9% [95%eCI: 0.9–4.7] in men). Older women reported the largest mortality fraction among all subgroups with 4.2% [95%eCI: 0.9–7.2] and more than 430 heat-related deaths.

### 3.4 Heat mortality attributable to human-induced climate change

**Figure 4:**
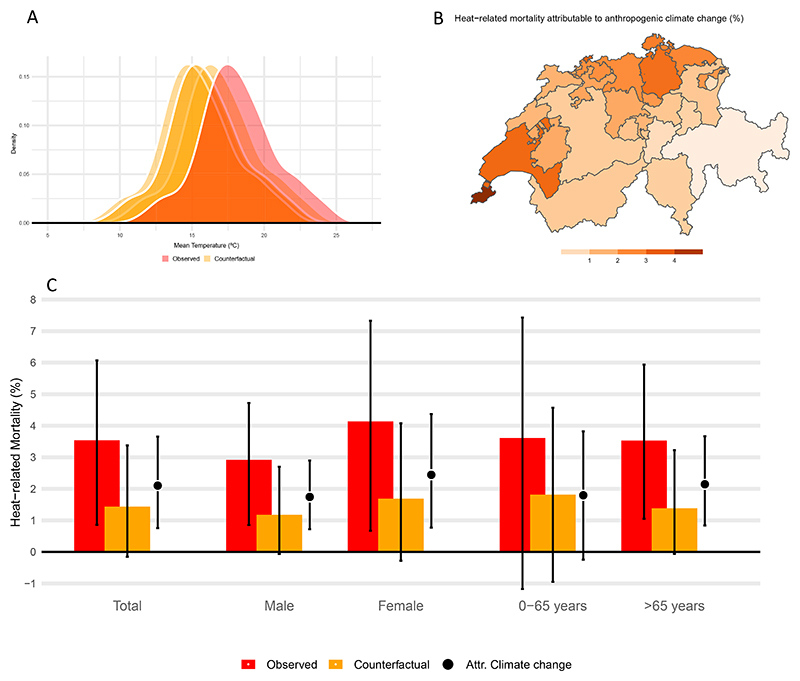
(A) Probability distribution of the observed daily mean temperature in summer 2022 (‘observed’) and the modelled series in the counterfactual scenario (‘counterfactual’). (B) Canton-specific heat-related mortality attributed to anthropogenic climate change, expressed as mortality fraction (%). (C) Heat-related mortality expressed as mortality fraction (%) over the all-cause deaths in the summer of 2022 estimated in the factual (‘observed’) and counterfactual scenario, and the difference between the two corresponds to the contribution of anthropogenic climate change. The vertical black bars depict the 95% empirical confidence interval.

Figure 4(A) shows the distribution of the observed daily mean temperature in Switzerland between June and August 2022 (averaged across cantons) and the resulting counterfactual series. According to the IPCC (2021), the observed global warming since 1850–1900 (1.15 °C) is attributable to human influence, with anthropogenic greenhouse gas emissions *very likely* being the main driver, and negligible natural sources of warming (based on the best estimates). We assumed here that the regional warming in Switzerland (1.19 °C–2.75 °C depending on model or observational data set) is also entirely anthropogenic, and subtracted it from the observed daily mean temperature series in each canton.

Figure 4(C) compares the observed heat-related mortality (in fraction, %) with the hypothetical burden in absence of climate change, depicted both with bars, and the resulting difference between the two scenarios, corresponding to the anthropogenic contribution, is displayed with the dot/segment. In absence of an anthropogenic signal, the heat-related burden would have amounted to 1.4% [95%eCI: *−*0.2 to 3.4] of all-cause mortality, corresponding to 253 deaths [95%eCI: *−*27 to 594]. Thus, 2.1% [95%eCI: 0.8–3.7] of the all-cause mortality in the summer of 2022 would have been avoided in absence of anthropogenic climate change. This corresponds to 370 [95%eCI: 133–644] deaths and 60% of the observed burden between June and August 2022. As in the observed burden, 60% of heat-related deaths attributed to climate change happened in females (220 [95%eCI: 69–393] vs. 150 [95%eCI: 62–250] in males), and 90% in older adults (330 [95%eCI: 129–565] vs. 39 [95%eCI: *−*5 to 84]). Again, older females seem to be the most affected with 2.5% [95%eCI: 0.9–1294.4] of all-cause mortality due to heat which can be attributed to climate change. We observe large differences in human-induced heat-related mortality fractions across cantons, with generally higher burden in urban cantons above 3% of all-cause mortality, in the cantons of Geneva, Vaud, Basel-Stadt and Zurich (figure 4(B), supplementary figure 3 shows the estimates by canton for the factual (observed) and counter-factual).

## 4 Discussion

The extreme heat during the summer of 2022 resulted in a substantial health burden amounting to more than 600 deaths due to heat in Switzerland, corresponding to 3% of all-cause deaths between June and August. Adults aged above 65 years were those affected the most, with nearly 90% of the observed heat-related deaths, and in particular, older women were the group most vulnerable to heat. The observed burden is three times the average annual heat-related deaths in Switzerland between 2009 and 2017 [25], which confirms this summer as one of the most devastating of the last decade in terms of heat-related mortality. We conclude based on the available evidence that the role of human-induced climate change as a driver of the extreme weather of the summer of 2022 in Europe is irrefutable [2, 4, 5]. In particular, according to observations and climate model simulations, the attributable warming in June–August mean temperatures across Switzerland ranges between 1.2 and 2.8 °C. This additional warming, resulting from anthropogenic activities, is responsible for nearly 60% of the observed heat-related mortality burden. This percentage is coherent with but higher than the results of a recent multi-regional assessment reporting a 37% of anthropogenic footprint across the last 30 years with an average increase in temperature of 0.9 °C in the studies locations [8]. This high attributable fraction reflects the exceptionality of this season in terms of unprecedented extreme weather, record-shattering events, and the growing footprint of climate change. But also, shows the consequences of the growing vulnerability of the population, despite adaptation efforts, due to co-existing public health challenges (i.e. the ongoing COVID-19 pandemic) and the increasing number of susceptible individuals at risk due to population ageing.

As for most of western and central Europe, the summer of 2022 was also exceptionally hot and dry in Switzerland. Average temperatures between June and August 2022 ranked second since the beginning of the records, exceeded only by 2003 [21]. According to a recent report, Geneva, Sion, and Lugano experienced 41, 49, and 38 hot days with maximum temperatures above 30 °C, respectively, the second-highest number in the observational record after 2003 [21]. The infamous summer of 2003 resulted in nearly 1000 excess deaths in Switzerland [22]. Although there are no published estimates on the mortality directly attributed to heat, one could argue that, given the magnitude of the event and the absence of adaptation measures at that time, heat may have been responsible for a substantial fraction of total excess mortality larger than the one for 2022 summer estimated in this study.

The method applied here combines the state-of-the-art approaches in epidemiology and attribution science widely used in individual disciplines. In particular, using high-resolution temperature and mortality data we performed a sophisticated epidemiological analysis to obtain robust estimates of mortality risk associated with heat in each canton and population subgroup [29, 30, 32]. In this way, we properly account for the different vulnerability to heat across Swiss subpopulations [25, 40]. These differences are mostly driven by climatic conditions (e.g. acclimatization), environmental features (e.g. urban profile), and characteristics of the population (e.g. socioeconomic status, demographic structure) [40, 41]. Additionally, given the current federal structure in this country different public health measures are currently in place in each canton [42]. For example, while soon after the hot summer of 2003 health authorities in the cantons of Geneva and Vaud implemented heat-health action plans (HHAPs), such a systematic and comprehensive public health response to heat is not available in Zurich and Basel-Stadt [42, 43]. In our assessment, we found large differences in heat-mortality impacts across cantons, being Geneva, Vaud, Basel-Stadt and Zurich the most affected. Consequently, the cantonal HHAPs may have prevented an even larger heat-related mortality burden, in particular in Geneva and Vaud where temperatures were particularly high during the 2022 summer (figure 2). Nevertheless, the elevated health burden estimated in these regions suggests that there is still a need for improvement, particularly in regions most affected by heat.

Additionally, to calculate the contribution of human-induced climate change we applied the method recently developed in a health attribution study [8], that builds upon an established approach to derive health impact projections [44]. The methodology applied here has been extensively described and discussed in Stuart-Smith *et al* [45]. Specifically, we adapted the approach used in Vicedo-Cabrera *et al* (2021) for trend attribution analysis to our study setting which considers a shorter period of time, similar to the event-attribution setting. In this analysis, we used observed mortality and temperature series, instead of modelled baseline mortality and simulated temperature series from global circulation models (GCMs) as in Vicedo-Cabrera *et al* (2021). We provide further details on these novel aspects in the following paragraphs. Previous impact attribution studies of extreme events applied an extension of the fraction of attributable risk probabilistic framework [9, 46]. This method compares the probability between scenarios of an ‘event class’ defined based on a fixed magnitude threshold. However, a recent publication raised some interpretability issues about such an approach [47]. Additionally, Stuart-Smith *et al* argues that the use of probability-based approaches would imply the simplification of the exposure-response curve (i.e. dichotomized exposure) and would disregard risks of moderate temperatures leading to an underestimation of the impacts. Thus, we consider our approach more suitable since it relies on standard epidemiological methods and the interpretation of the findings would be less prone to confusion.

The definition of the counterfactual scenarios (i.e. temperature series without anthropogenic climate change) is one of the key steps of attribution analysis. We derived the counterfactual series by subtracting the anthropogenic temperature change (estimated from observations and models) from the observed daily temperature, assuming that the attributable warming is homogeneous across Switzerland. Potentially amplified warming at high elevation has a negligible effect here since temperatures are population-weighted with very little weights at high elevation. The range of attributable warming of 1.2 °C and 2.8 °C represents the full uncertainty in observations and models, with the observations being at the high end of model estimates. According to the best estimate in IPCC (2021), the entire global warming since 1850–1900 is attributable to human influence [18]. We here assume that the regional warming in Switzerland directly scales with the global warming, an assumption that is generally verified in most regions both in observations and models [48–50]. The observed warming may be further affected by unforced internal variability, however, note that the uncertainty range here includes a set of CMIP6 (GCMs) with different realizations of variability. Overall, we consider our uncertainty estimate covering model uncertainties, internal variability, as well as observational uncertainty as a conservative estimate of the uncertainty in attributable warming.

In the attribution analysis, we used observed temperature and mortality series to derive the anthropogenic impacts. This approach differs from the one of Vicedo-Cabrera *et al* (2022) which used pairs of bias-corrected temperature series from climate models (i.e. GCM) and average baseline mortality—similar to health impact projections [8, 44]. The use of observed series presents several advantages compared to GCM-based series. Specifically, this approach captures the observed day-to-day variations and thereby provides more accurate estimates of heat impacts, especially in short periods or events, as is the case of this study, and more reliable absolute measures of impacts (i.e. the number of deaths attributed to heat). The main reason for this is that this approach better captures the short-term variation in mortality associated with temperature since both temperature and mortality are aligned in time. We acknowledge that it is uncertain how the temporal trends of observed mortality would have been in absence of anthropogenic climate change. However, we believe that under the current study setting (i.e. short period and scenarios based on observed temperatures) assuming the same temporal trends as in the factual scenario would be more reliable for the reasons stated above. To note, freely running fully-coupled GCMs produce a random realization of day-to-day and year-to-year variations consistent with the long-term climate pattern but do not coincide with the daily variation of observed temperatures and mortality within an observed event. Thus, the use of daily average mortality in that case, instead of the observed, would be more advisable, but it might lead to an underestimation of the mortality burden when the method is applied to short periods (i.e. events) [45].

The unique circumstances of the summer of 2022 derived from the still ongoing COVID-19 pandemic led to several complexities in the modelling approach and additional uncertainties on the causal link between heat and mortality. First, we used heat-mortality associations estimated between 1990 and 2017 to derive the health impacts in 2022, since we did not have access to individual mortality data after 2017. Also, it remains unclear whether mortality data in 2020 and 2021 can be reliably used in this kind of assessments since COVID-related mortality waves could introduce biases in the assessment of health risks associated with time-varying factors during this period (e.g. harvesting). We, thus, assume that vulnerability in 2022 did not change with regard to the average mortality risks between 1990 and 2017. This may be a strong assumption given that several cantonal HHAPs have implemented new measures (e.g. the canton of Zurich) between 2018 and 2021 which could have presumably reduced their vulnerability [51]. However, we consider ours a more sensible, and possibly conservative approach since the gap is relatively small in comparison with the length of the whole series (i.e. nearly 30 years) which would allow us to derive robust risk estimates. Second, we deliberately disregarded the presence of COVID-19 mortality during the summer of 2022 when estimating the mortality attributed to heat. It should be noted that the cause of death cannot be used to determine the real magnitude of heat or other environmental factors (e.g. air pollution) since these are typically underreported. In the case of COVID-19 deaths, the successive changes in case definition and reporting criteria may also lead to strong biases [52]. As shown in the directed acyclic graph in supplementary figure 4, we consider that COVID-19 may act as a mediator between heat and mortality by altering the population’s vulnerability. COVID-19 may have increased the population at risk (i.e. infected population or with sequelae of previous SARS-CoV2 infection) and the vulnerability of the already frail population (i.e. overwhelmed healthcare system) [53]. Heat stress can also compromise the immune system and, thus, increase the severity of COVID-19 symptoms [54]. Additionally, recent studies seem to indicate that weather can also alter transmissibility patterns of SARS-CoV2, although evidence remains sparse [55, 56]. Thus, it implies that our estimates of the mortality burden would account for the direct and indirect effects of heat partly amplified by the COVID-19 pandemic during the summer of 2022.

The exceptional heat in the summer of 2022 resulted in a substantial mortality burden in Switzerland, despite the adaptation measures implemented during the last decade. More importantly, our findings indicate that anthropogenic climate change is responsible for more than half of the observed burden. This suggests that the anthropogenic foot-print in the observed climate-sensitive health burden may be steeply increasing as warming progresses. Thus, ambitious mitigation efforts and adaptation strategies are urgently needed since the summer of 2022, considered amongst the warmest in recent times, is expected to be one of the coolest seasons in the future under climate change emission scenarios.

## Supplementary Material

Supplementary data

## Data Availability

The data that support the findings of this study are openly available at the following URL/DOI: https://doi.org/10.48620/315.

## You may also like

- Impacts of hot-dry conditions on hydropower production in Switzerland Noelia Otero, Pascal Horton, Olivia Martius et al.
- Derivation of new diagnostic reference levels for neuro-paediatric computed tomography examinations in Switzerland Franca Wagner, Julie Bize, Damien Racine et al.
- Relationships between coworking spaces and CO_2_ emissions in work-related commuting: first empirical insights for the case of Switzerland with regard to urban-rural differences Timo Ohnmacht, Jana Z’Rotz and Lisa Dang

## Code availability

R Code is publicly available on GitHub of the first author of the publication (https://github.com/anavica/ERL_heatmortality2022.git).
