# Supplementary material for: The footprint of human-induced climate change on heat-related deaths in the summer of 2022 in Switzerland
Source: Environ Res Lett. Author manuscript; Available in PMC 2024 Mar 12. (PMC7615730; doi:10.1088/1748-9326/ace0d0)
Supplement: Supplementary data [file EMS194268-supplement-Supplementary_data.docx]

**SUPPLEMENTARY MATERIAL**

*" The footprint of human-induced climate change on heat-related deaths in the summer of 2022 in Switzerland " Vicedo-Cabrera et al.*

**Suppl. Methods 1. Estimation of the exposure-response association.**

First stage. The case time series design

The modelling framework is defined based on the following formula:


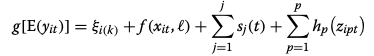


The main difference from the traditional time series is the presence of multiple series represented by $i$. For each canton and combination of sex and age subgroups, $y_{it}$ represents daily all-cause death counts for each of the $i=1,...I$ municipalities in each canton. The temperature-mortality association is modelled through a distributed-lag non-linear model (DLNM) with a cross-basis term $f(x_{it}, l)$. This is a bi-dimensional term defining the (1) exposure-response dimension with a natural spline and two internal knots at the 50^th^ and 90^th^ percentile, and the (2) lag-response dimension (i.e., distribution of the association across lags) with a natural spline and two internal knots equally spaced in the log scale and considering 10 days of lag. This parametrization has been extensively used in previous multi-location assessments (1). Different to the traditional time-series regression, we define matched *risk sets* with intercepts $\varepsilon_{i(k)}$ expressing baseline risks varying across observational units $i$and time stratum $k$. This time stratus is defined with combinations of year/month/day of the week strata indicators within municipalities $i$. Using this matching risk sets we can efficiently control for long-term and seasonal patterns. For example, the first Monday in June 2009 will be compared to all other Mondays of the same month and year within each municipality. The model is fitted using a conditional quasi-Poisson regression (i.e., accounting for overdispersion).

Second stage. Multivariate multilevel meta-analysis

For each combination of sex and age subgroups, we performed a random-effects multivariate multilevel meta-analysis of the canton-specific exposure-response associations estimated in the previous step (2). Specifically, we derived the reduced sets of coefficients and covariance matrices (i.e., into uni-dimensional parameters) defining the overall cumulative exposure-response association across 10 days of lag. Following the procedure in De Schrijver et al. (2022), we included as a fixed-effects predictor the average mean temperature per canton, and a random effects component by region (i.e., seven big regions in Switzerland) to allow variability across cantons within each regional cluster. We derive the pooled overall cumulative curve representing the average temperature-mortality association, and the best linear unbiased predictors (BLUPs) for each canton, which are improved estimates of the temperature-mortality association (2). We report the heat-mortality association in terms of relative risks (RR) and the corresponding 95% confidence intervals (CI) at each temperature value above the temperature of minimum mortality (MMT), used here as a reference. MMT can be considered the optimum temperature corresponding to the minimum mortality risk (3). This value was restricted between the 25th and 90th percentile of the temperature distribution, as done in previous work (1). This restriction allows us to obtain more reliable risk estimates, in particular in small locations with low statistical power where the curve could be very wiggly in the extremes and thus, leading to extreme MMT values and very unprecise risk estimates. Additionally, by applying this restriction, we limit the MMT in temperatures that are within a reasonable range for which we could consider "heat" above them (i.e., above the 25^th^ percentile).

**Suppl. Methods 2. Definition of the counterfactual temperature series**

To compute the counterfactual temperature time series for the summer of 2022 in Switzerland, we first estimate the mean human-induced warming level for June—August across the entire country. For this, we rely on both observations and climate model simulations of daily mean temperatures averaged over Switzerland. The observational datasets include gridded E-OBS data v25.e and an area-averaged temperature time series from MeteoSwiss (4–6), with the latter being based on the homogenized station as well as high-resolution gridded data. Global mean surface temperatures (GMSTs) are taken from HadCRUT5 (7). We also employ historical CMIP6 simulations extended in the last few years with the SSP5-8.5 scenario (8), extracting the summertime daily mean temperature over Switzerland and GMST for a total of 25 models, always using the first available ensemble member.

The warming level is estimated by linearly regressing average Swiss summertime temperatures on GMST, either combining observations (MeteoSwiss or E-OBS and HadCRUT5) or using only climate model output (i.e., we infer the (linear) relationship between mean June—August temperatures in Switzerland and global warming). HadCRUT5 and all respective CMIP6 GMSTs are smoothed with a 4-year running mean prior to analysis. Due to HadCRUT5 data availability constraints, MeteoSwiss temperatures are evaluated from 1864 to 2022, and the same period is chosen for all CMIP6 models, whereas E-OBS only extends back to 1920. The resulting slopes from the linear regressions inform on the warming of Swiss summers (in [°](https://www.degreesymbol.net/)C) per [°](https://www.degreesymbol.net/)C GMST increase, and we convert these slopes to warming levels by multiplying with a GMST increase of 1.15 [°](https://www.degreesymbol.net/)C attributable to human influence (IPCC 2021 SPM or chapter 3). By doing so, we consider that the pace of global warming varies across climate models. This results in a warming level of 2.16 [°](https://www.degreesymbol.net/)C according to E-OBS, 2.75 [°](https://www.degreesymbol.net/)C with MeteoSwiss data, and a range of 1.19 to 2.27 [°](https://www.degreesymbol.net/)C based on the CMIP6 models, calculated as the intermodel mean +/- 1 standard deviation. We derive 4 counterfactual temperature series between June—August 2022 by subtracting these 4 warming levels from the population-weighted daily mean temperature in 2022 for each canton.

**References**

1. Vicedo-Cabrera AM, Scovronick N, Sera F, Royé D, Schneider R, Tobias A, et al. The burden of heat-related mortality attributable to recent human-induced climate change. Nat Clim Chang. 2021 Jun;11(6):492–500.

2. Sera F, Armstrong B, Blangiardo M, Gasparrini A. An extended mixed-effects framework for meta-analysis. Statistics in Medicine. 2019;38(29):5429–44.

3. Gasparrini A, Guo Y, Hashizume M, Lavigne E, Zanobetti A, Schwartz J, et al. Mortality risk attributable to high and low ambient temperature: a multicountry observational study. Lancet. 2015 Jul 25;386(9991):369–75.

4. Cornes RC, van der Schrier G, van den Besselaar EJM, Jones PD. An Ensemble Version of the E-OBS Temperature and Precipitation Data Sets. J Geophys Res Atmos. 2018 Sep 16;123(17):9391–409.

5. Begert M, Frei C. Long-term area-mean temperature series for Switzerland-Combining homogenized station data and high resolution grid data. Int J Climatol. 2018 May;38(6):2792–807.

6. MeteoSchweiz. CHTM: Area-mean temperature of Switzerland [Internet]. Federal Office of Meteorology and Climatology MeteoSwiss; 2022 [cited 2023 Jan 23]. Available from: https://www.meteoswiss.admin.ch/climate/climate-change/changes-in-temperature-precipitation-and-sunshine/swiss-temperature-mean/data-on-the-swiss-temperature-mean.html

7. Morice CP, Kennedy JJ, Rayner NA, Winn JP, Hogan E, Killick RE, et al. An Updated Assessment of Near‐Surface Temperature Change From 1850: The HadCRUT5 Data Set. Geophys Res Atmos [Internet]. 2021 Feb 16 [cited 2023 Jan 23];126(3). Available from: https://onlinelibrary.wiley.com/doi/10.1029/2019JD032361

8. Eyring V, Bony S, Meehl GA, Senior CA, Stevens B, Stouffer RJ, et al. Overview of the Coupled Model Intercomparison Project Phase 6 (CMIP6) experimental design and organization. Geoscientific Model Development. 2016 May 26;9(5):1937–58.

**Suppl. Table 1.** Description of the temperature and mortality data in June-August between 1990-2017. Number of all-cause deaths (N) per sex and age category in each canton. Average and range of the daily mean temperature by canton (population-weighted average).

|  | **All-cause mortality (N)** | | | | **Mean temperature** |
| --- | --- | --- | --- | --- | --- |
|  | Male 0-65 years | Male >65 years | Female 0-65 years | Female >65 years | Average [range] |
| *Zurich* | 7069 | 17649 | 4265 | 18691 | 18.0 [3.5;28.2] |
| *Bern* | 6358 | 20467 | 3289 | 22408 | 16.5 [-6.1;28.3] |
| *Luzern* | 2138 | 6234 | 1116 | 6771 | 17.2 [0.2;28.0] |
| *Uri* | 210 | 872 | 99 | 846 | 11.0 [-5.6;27.6] |
| *Schwyz* | 782 | 2369 | 406 | 2555 | 15.6 [-1.2;27.4] |
| *Obwalden* | 171 | 718 | 88 | 688 | 13.1 [-2.8;25.9] |
| *Nidwalden* | 212 | 697 | 119 | 703 | 15.4 [-0.6;27.4] |
| *Glarus* | 292 | 890 | 125 | 1008 | 12.2 [-2.3;23.1] |
| *Zug* | 554 | 1556 | 312 | 1791 | 17.1 [3.3;28.0] |
| *Fribourg* | 1808 | 4671 | 854 | 4502 | 17.0 [-0.0;28.3] |
| *Solothurn* | 1656 | 5330 | 928 | 5919 | 17.7 [3.7;28.8] |
| *Basel-Stadt* | 1161 | 2706 | 792 | 3156 | 19.0 [7.1;29.3] |
| *Basel-Landschaft* | 1526 | 5090 | 899 | 5492 | 18.0 [3.7;29.2] |
| *Schaffhausen* | 466 | 1637 | 288 | 1909 | 17.8 [4.9;27.8] |
| *Appenzell Ausserrhoden* | 340 | 1201 | 182 | 1400 | 16.1 [2.2;27.6] |
| *Appenzell Innerrhoden* | 92 | 340 | 54 | 337 | 15.2 [0.6;27.2] |
| *St. Gallen* | 2897 | 8671 | 1526 | 9652 | 16.8 [-1.1;28.6] |
| *Graubunden* | 1288 | 4064 | 607 | 4369 | 12.1 [-5.3;27.2] |
| *Aargau* | 3520 | 10091 | 1839 | 11081 | 18.1 [5.1;28.8] |
| *Thurgau* | 1487 | 4335 | 793 | 4957 | 17.9 [5.1;28.2] |
| *Ticino* | 1968 | 6354 | 1064 | 7488 | 18.3 [-3.8;29.8] |
| *Vaud* | 4073 | 11706 | 2263 | 13166 | 17.8 [-1.8;28.8] |
| *Valais* | 2141 | 5672 | 1021 | 5640 | 12.6 [-7.2;28.1] |
| *Neuchatel* | 1226 | 3514 | 635 | 4137 | 16.5 [2.2;28.6] |
| *Geneva* | 2420 | 5674 | 1443 | 6761 | 19.4 [8.4;29.6] |
| *Jura* | 526 | 1579 | 235 | 1683 | 16.8 [2.8;29.2] |

**Suppl. Table 2.** Mortality risk associated with heat in Switzerland and each canton by sex-age groups between 1990-2017 (June-August). These are defined as the relative risk (RR, and 95% confidence interval (CI)) at the 99th percentile of the summer temperature distribution, with reference to the temperature of minimum mortality (MMT, corresponding percentile denoted as MMP).

|  | **Male 0-65 years** | | **Male >65 years** | | **Female 0-65 years** | | **Female >65 years** | |
| --- | --- | --- | --- | --- | --- | --- | --- | --- |
|  | **MMT (MMP)** | **Heat-RR [95% CI]** | **MMT (MMP)** | **Heat-RR [95% CI]** | **MMT (MMP)** | **Heat-RR [95% CI]** | **MMT (MMP)** | **Heat-RR [95% CI]** |
| *Pooled* | 18.4 (69) | 1.15 [0.99 - 1.33] | 18.5 (70) | 1.23 [1.13 - 1.33] | 15.5 (41) | 1.08 [0.90 - 1.31] | 13.6 (25) | 1.36 [1.22 - 1.51] |
| *Zürich* | 15.8 (27) | 1.18 [1.00 - 1.40] | 15.6 (25) | 1.30 [1.18 - 1.42] | 20.5 (74) | 1.18 [0.88 - 1.57] | 15.6 (25) | 1.41 [1.21 - 1.65] |
| *Bern* | 18.8 (69) | 1.19 [1.05 - 1.35] | 19.1 (72) | 1.22 [1.14 - 1.31] | 14.3 (28) | 1.06 [0.91 - 1.24] | 14.5 (29) | 1.17 [1.04 - 1.33] |
| *Luzern* | 20.7 (82) | 1.12 [0.96 - 1.31] | 19.1 (67) | 1.25 [1.15 - 1.36] | 14.7 (25) | 1.09 [0.90 - 1.32] | 14.7 (25) | 1.42 [1.20 - 1.69] |
| *Uri* | 17.0 (90) | 0.94 [0.63 - 1.40] | 17.0 (90) | 1.06 [0.87 - 1.30] | 10.7 (46) | 1.23 [0.64 - 2.34] | 13.8 (72) | 1.29 [1.01 - 1.65] |
| *Schwyz* | 20.3 (88) | 1.12 [0.94 - 1.33] | 18.5 (75) | 1.21 [1.10 - 1.34] | 14.1 (35) | 1.07 [0.84 - 1.36] | 12.9 (25) | 1.39 [1.17 - 1.66] |
| *Obwalden* | 18.6 (90) | 0.96 [0.63 - 1.48] | 17.2 (82) | 1.13 [0.89 - 1.44] | 12.1 (40) | 1.09 [0.58 - 2.07] | 15.1 (66) | 1.42 [1.09 - 1.87] |
| *Nidwalden* | 20.2 (88) | 1.17 [0.98 - 1.39] | 18.5 (76) | 1.23 [1.11 - 1.36] | 14.3 (39) | 1.05 [0.82 - 1.34] | 12.6 (25) | 1.41 [1.18 - 1.69] |
| *Glarus* | 13.0 (55) | 0.75 [0.31 - 1.81] | 15.3 (76) | 1.08 [0.67 - 1.75] | 11.4 (41) | 1.07 [0.30 - 3.75] | 15.2 (75) | 1.46 [0.90 - 2.39] |
| *Zug* | 21.1 (85) | 1.18 [1.04 - 1.35] | 19.4 (71) | 1.26 [1.17 - 1.35] | 15.6 (34) | 1.07 [0.91 - 1.27] | 14.5 (25) | 1.41 [1.19 - 1.67] |
| *Fribourg* | 16.0 (38) | 1.14 [0.98 - 1.34] | 19.2 (70) | 1.20 [1.11 - 1.30] | 14.8 (27) | 1.11 [0.92 - 1.34] | 16.1 (39) | 1.18 [1.04 - 1.35] |
| *Solothurn* | 17.0 (42) | 1.13 [0.93 - 1.38] | 15.3 (25) | 1.18 [1.06 - 1.31] | 15.6 (28) | 1.17 [0.91 - 1.49] | 17.4 (46) | 1.18 [1.02 - 1.36] |
| *Basel-Stadt* | 16.5 (25) | 1.37 [1.10 - 1.70] | 16.5 (25) | 1.36 [1.20 - 1.53] | 16.5 (25) | 1.13 [0.84 - 1.53] | 20.8 (69) | 1.42 [1.19 - 1.69] |
| *Basel-Land.* | 15.4 (25) | 1.28 [1.09 - 1.51] | 19.8 (68) | 1.30 [1.19 - 1.43] | 15.4 (25) | 1.09 [0.89 - 1.35] | 20.0 (70) | 1.40 [1.20 - 1.63] |
| *Schaffhausen* | 16.7 (38) | 1.20 [1.00 - 1.43] | 15.3 (25) | 1.30 [1.18 - 1.43] | 19.3 (64) | 1.16 [0.88 - 1.53] | 20.1 (72) | 1.34 [1.14 - 1.59] |
| *App. Auss.* | 18.8 (74) | 1.24 [1.05 - 1.46] | 18.3 (70) | 1.28 [1.17 - 1.40] | 19.5 (80) | 1.12 [0.84 - 1.50] | 18.7 (73) | 1.32 [1.13 - 1.55] |
| *App. Innerr.* | 18.8 (79) | 1.23 [1.00 - 1.51] | 18.0 (73) | 1.27 [1.13 - 1.42] | 18.4 (76) | 1.11 [0.79 - 1.55] | 18.0 (73) | 1.33 [1.12 - 1.58] |
| *St. Gallen* | 19.4 (72) | 1.26 [1.08 - 1.47] | 18.8 (67) | 1.27 [1.17 - 1.38] | 20.8 (83) | 1.14 [0.87 - 1.48] | 19.4 (72) | 1.28 [1.11 - 1.47] |
| *Graubünden* | 17.8 (88) | 1.15 [0.82 - 1.63] | 16.8 (83) | 1.18 [0.97 - 1.44] | 13.7 (63) | 1.05 [0.63 - 1.75] | 15.6 (76) | 1.28 [1.03 - 1.60] |
| *Aargau* | 16.6 (34) | 1.14 [0.96 - 1.37] | 15.7 (25) | 1.23 [1.11 - 1.35] | 15.8 (26) | 1.15 [0.93 - 1.41] | 20.3 (72) | 1.34 [1.17 - 1.55] |
| *Thurgau* | 17.2 (42) | 1.14 [0.93 - 1.40] | 15.6 (25) | 1.23 [1.11 - 1.37] | 16.4 (33) | 1.16 [0.90 - 1.48] | 20.3 (74) | 1.27 [1.09 - 1.47] |
| *Ticino* | 21.7 (78) | 1.39 [1.01 - 1.92] | 20.8 (69) | 1.28 [1.08 - 1.51] | 22.9 (88) | 1.12 [0.72 - 1.75] | 15.7 (25) | 1.39 [1.12 - 1.71] |
| *Vaud* | 15.3 (25) | 1.33 [1.14 - 1.56] | 19.6 (66) | 1.28 [1.17 - 1.39] | 15.3 (25) | 1.06 [0.87 - 1.30] | 15.3 (25) | 1.47 [1.27 - 1.69] |
| *Valais* | 17.9 (85) | 1.38 [0.99 - 1.91] | 17.6 (83) | 1.23 [1.02 - 1.49] | 13.8 (58) | 0.96 [0.61 - 1.52] | 9.2 (25) | 1.43 [1.15 - 1.80] |
| *Neuchâtel* | 20.2 (81) | 1.30 [1.08 - 1.57] | 19.7 (77) | 1.26 [1.14 - 1.40] | 19.9 (79) | 1.04 [0.78 - 1.39] | 16.0 (44) | 1.19 [1.02 - 1.37] |
| *Geneva* | 18.4 (38) | 1.37 [1.09 - 1.71] | 17.1 (25) | 1.35 [1.19 - 1.52] | 17.1 (25) | 1.15 [0.87 - 1.53] | 17.8 (32) | 1.54 [1.28 - 1.84] |
| *Jura* | 16.9 (51) | 1.15 [0.99 - 1.34] | 19.5 (75) | 1.20 [1.12 - 1.28] | 15.9 (41) | 1.10 [0.94 - 1.30] | 16.5 (47) | 1.18 [1.04 - 1.34] |

**Suppl. Table 3.** Description of the temperature and mortality data in June-August 2022. Number of all-cause deaths (N) per sex and age category in each canton. Average and range of the daily mean temperature by canton (population-weighted average).

|  | **All-cause mortality (N)** | | | | **Mean temperature** |
| --- | --- | --- | --- | --- | --- |
|  | Male 0-65 years | Male >65 years | Female 0-65 years | Female >65 years | Average [range] |
| *Zurich* | 202 | 1191 | 129 | 1327 | 20.2 [13.4;27.0] |
| *Bern* | 186 | 977 | 104 | 1146 | 17.3 [11.3;23.3] |
| *Luzern* | 70 | 340 | 32 | 391 | 18.9 [11.8;26.1] |
| *Uri* | 6 | 19 | 4 | 26 | 13.0 [7.9;19.3] |
| *Schwyz* | 27 | 128 | 13 | 128 | 17.1 [10.2;23.6] |
| *Obwalden* | 7 | 26 | 2 | 32 | 16.1 [8.9;22.6] |
| *Nidwalden* | 6 | 38 | 5 | 51 | 17.1 [10.1;24.3] |
| *Glarus* | 6 | 27 | 5 | 40 | 14.5 [8.2;20.6] |
| *Zug* | 18 | 78 | 10 | 93 | 19.3 [12.4;26.4] |
| *Fribourg* | 48 | 254 | 46 | 250 | 19.0 [11.4;25.6] |
| *Solothurn* | 47 | 264 | 29 | 269 | 19.6 [13.1;26.2] |
| *Basel-Stadt* | 46 | 200 | 21 | 250 | 21.6 [15.8;28.7] |
| *Basel-Landschaft* | 48 | 314 | 28 | 324 | 20.4 [14.0;27.6] |
| *Schaffhausen* | 14 | 88 | 9 | 98 | 20.1 [12.9;26.5] |
| *Appenzell Ausserrhoden* | 12 | 51 | 5 | 62 | 17.7 [10.4;25.8] |
| *Appenzell Innerrhoden* | 6 | 18 | 2 | 21 | 16.9 [9.3;25.0] |
| *St. Gallen* | 100 | 411 | 48 | 467 | 18.0 [11.2;25.3] |
| *Graubunden* | 32 | 207 | 17 | 248 | 13.0 [8.1;18.1] |
| *Aargau* | 106 | 555 | 70 | 555 | 20.4 [13.8;27.1] |
| *Thurgau* | 47 | 235 | 36 | 243 | 20.1 [13.2;26.8] |
| *Ticino* | 54 | 344 | 33 | 406 | 17.8 [13.2;21.7] |
| *Vaud* | 119 | 598 | 74 | 733 | 19.8 [12.0;25.9] |
| *Valais* | 77 | 320 | 27 | 333 | 14.1 [9.3;19.1] |
| *Neuchatel* | 30 | 135 | 23 | 205 | 18.4 [11.2;24.9] |
| *Geneva* | 56 | 344 | 43 | 401 | 22.3 [14.1;28.6] |
| *Jura* | 10 | 71 | 2 | 75 | 19.0 [12.3;26.4] |

**Suppl. Table 4.** Heat-related mortality between June-August 2022. "Observed" corresponds to the burden in the factual scenario (with climate change), "counterfactual" is the burden in the scenario without climate change, "difference" is the difference between the two scenarios corresponding to the burden attributable to human-induced climate change, "prop" is the proportion of observed heat-related deaths attributable to human-induced climate change. N, number of deaths; %, fraction over all-cause summer mortality.

|  | **N observed** | **N counterfct** | **N difference** | **% observed** | **% counterfct** | **% difference** | **% prop** |
| --- | --- | --- | --- | --- | --- | --- | --- |
| *Total* | 623 [151;1069] | 253 [-27;594] | 370 [133;644] | 3.5 [0.9;6.1] | 1.4 [-0.2;3.4] | 2.1 [0.8;3.7] | 59.3 |
| *Male 0-65years* | 48 [-0;87] | 22 [-3;51] | 25 [1;50] | 3.4 [-0.0;6.3] | 1.6 [-0.2;3.7] | 1.8 [0.1;3.6] | 52.8 |
| *Male >65years* | 204 [73;326] | 79 [-4;182] | 125 [57;202] | 2.8 [1.0;4.5] | 1.1 [-0.1;2.5] | 1.7 [0.8;2.8] | 61.3 |
| *Female 0-65years* | 32 [-25;77] | 17 [-18;50] | 14 [-8;35] | 3.9 [-3.1;9.4] | 2.1 [-2.2;6.1] | 1.8 [-1.0;4.3] | 45.1 |
| *Female >65years* | 340 [73;591] | 135 [-14;324] | 205 [70;361] | 4.2 [0.9;7.2] | 1.6 [-0.2;4.0] | 2.5 [0.9;4.4] | 60.4 |
| *Male* | 251 [73;407] | 101 [-6;233] | 150 [62;250] | 2.9 [0.9;4.7] | 1.2 [-0.1;2.7] | 1.7 [0.7;2.9] | 59.7 |
| *Female* | 372 [60;659] | 152 [-25;366] | 220 [69;393] | 4.1 [0.7;7.3] | 1.7 [-0.3;4.1] | 2.4 [0.8;4.4] | 59.1 |
| *0-65years* | 79 [-26;163] | 40 [-21;100] | 39 [-5;84] | 3.6 [-1.2;7.4] | 1.8 [-0.9;4.6] | 1.8 [-0.2;3.8] | 49.7 |
| *>65years* | 544 [162;915] | 213 [-10;497] | 330 [129;565] | 3.5 [1.1;5.9] | 1.4 [-0.1;3.2] | 2.1 [0.8;3.7] | 60.8 |
| *Zürich* | 149 [34;255] | 61 [-8;145] | 88 [35;149] | 5.2 [1.2;8.9] | 2.2 [-0.3;5.1] | 3.1 [1.2;5.2] | 58.8 |
| *Bern* | 34 [-13;75] | 11 [-12;37] | 23 [-1;49] | 1.4 [-0.5;3.1] | 0.5 [-0.5;1.5] | 0.9 [-0.1;2.0] | 67.0 |
| *Luzern* | 22 [-3;44] | 7 [-7;24] | 15 [3;28] | 2.7 [-0.4;5.3] | 0.9 [-0.8;2.8] | 1.8 [0.4;3.4] | 66.3 |
| *Uri* | 0 [-0;1] | 0 [-0;0] | 0 [-0;1] | 0.6 [-0.7;1.8] | 0.1 [-0.3;0.6] | 0.5 [-0.5;1.5] | 85.4 |
| *Schwyz* | 5 [-2;11] | 2 [-2;5] | 3 [-0;7] | 1.6 [-0.6;3.8] | 0.5 [-0.6;1.9] | 1.1 [-0.0;2.4] | 68.2 |
| *Obwalden* | 1 [-1;3] | 1 [-0;1] | 1 [-0;2] | 1.9 [-1.1;4.5] | 0.8 [-0.5;2.2] | 1.2 [-0.7;3.0] | 59.6 |
| *Nidwalden* | 2 [-1;5] | 1 [-1;3] | 1 [-0;3] | 2.2 [-1.1;5.2] | 0.7 [-1.0;2.7] | 1.5 [-0.2;3.3] | 68.2 |
| *Glarus* | 1 [-3;3] | 0 [-1;1] | 1 [-2;2] | 1.0 [-3.8;4.4] | 0.2 [-1.4;1.8] | 0.8 [-2.4;3.1] | 78.1 |
| *Zug* | 7 [1;12] | 2 [-1;7] | 4 [1;8] | 3.4 [0.3;6.3] | 1.2 [-0.4;3.3] | 2.2 [0.6;4.0] | 63.6 |
| *Fribourg* | 18 [-0;33] | 7 [-3;18] | 10 [2;20] | 2.9 [-0.0;5.5] | 1.2 [-0.5;3.1] | 1.7 [0.4;3.3] | 59.1 |
| *Solothurn* | 19 [-8;43] | 8 [-7;24] | 11 [-2;25] | 3.2 [-1.3;7.0] | 1.3 [-1.1;3.9] | 1.9 [-0.3;4.2] | 59.0 |
| *Basel-Stadt* | 31 [13;47] | 14 [2;28] | 17 [8;28] | 6.0 [2.6;9.1] | 2.6 [0.4;5.3] | 3.3 [1.5;5.4] | 56.1 |
| *Basel-Landschaft* | 27 [14;39] | 10 [3;20] | 17 [8;26] | 3.8 [2.0;5.4] | 1.4 [0.4;2.9] | 2.4 [1.2;3.7] | 63.3 |
| *Schaffhausen* | 9 [4;14] | 3 [0;7] | 6 [3;9] | 4.3 [2.0;6.5] | 1.6 [0.2;3.5] | 2.8 [1.3;4.4] | 63.7 |
| *Ap. Ausserrhoden* | 5 [3;7] | 2 [1;4] | 3 [1;4] | 3.5 [2.0;5.1] | 1.4 [0.5;2.7] | 2.2 [1.0;3.4] | 61.5 |
| *Ap. Innerrhoden* | 0 [0;1] | 0 [-0;0] | 0 [0;1] | 0.7 [0.0;1.3] | 0.1 [-0.0;0.4] | 0.6 [0.1;1.1] | 83.5 |
| *St. Gallen* | 15 [7;22] | 4 [0;10] | 11 [5;17] | 1.4 [0.7;2.1] | 0.4 [0.0;1.0] | 1.1 [0.5;1.6] | 74.2 |
| *Graubünden* | 0 [-1;2] | 0 [-0;0] | 0 [-1;2] | 0.1 [-0.3;0.4] | 0.0 [-0.1;0.1] | 0.1 [-0.2;0.3] | 94.0 |
| *Aargau* | 52 [13;88] | 21 [-5;50] | 31 [13;52] | 4.1 [1.0;6.8] | 1.6 [-0.4;3.9] | 2.4 [1.0;4.0] | 60.1 |
| *Thurgau* | 23 [7;38] | 9 [-1;22] | 14 [7;23] | 4.2 [1.2;6.8] | 1.6 [-0.2;3.9] | 2.6 [1.2;4.1] | 61.5 |
| *Ticino* | 11 [-9;28] | 4 [-3;13] | 7 [-6;20] | 1.3 [-1.0;3.4] | 0.5 [-0.4;1.6] | 0.9 [-0.7;2.4] | 65.8 |
| *Vaud* | 87 [33;134] | 38 [2;80] | 49 [24;77] | 5.7 [2.2;8.8] | 2.5 [0.1;5.3] | 3.2 [1.6;5.1] | 56.4 |
| *Valais* | 14 [-14;38] | 6 [-9;22] | 8 [-6;20] | 1.8 [-1.9;5.0] | 0.8 [-1.2;2.8] | 1.0 [-0.8;2.7] | 55.3 |
| *Neuchâtel* | 9 [-0;17] | 3 [-1;8] | 6 [1;11] | 2.2 [-0.1;4.4] | 0.8 [-0.4;2.2] | 1.4 [0.2;2.8] | 65.2 |
| *Geneva* | 78 [38;116] | 38 [10;73] | 40 [18;64] | 9.3 [4.4;13.8] | 4.5 [1.2;8.6] | 4.8 [2.2;7.6] | 51.3 |
| *Jura* | 4 [1;7] | 2 [-0;4] | 2 [1;4] | 2.7 [0.4;4.7] | 1.2 [-0.1;2.7] | 1.5 [0.4;2.8] | 57.0 |


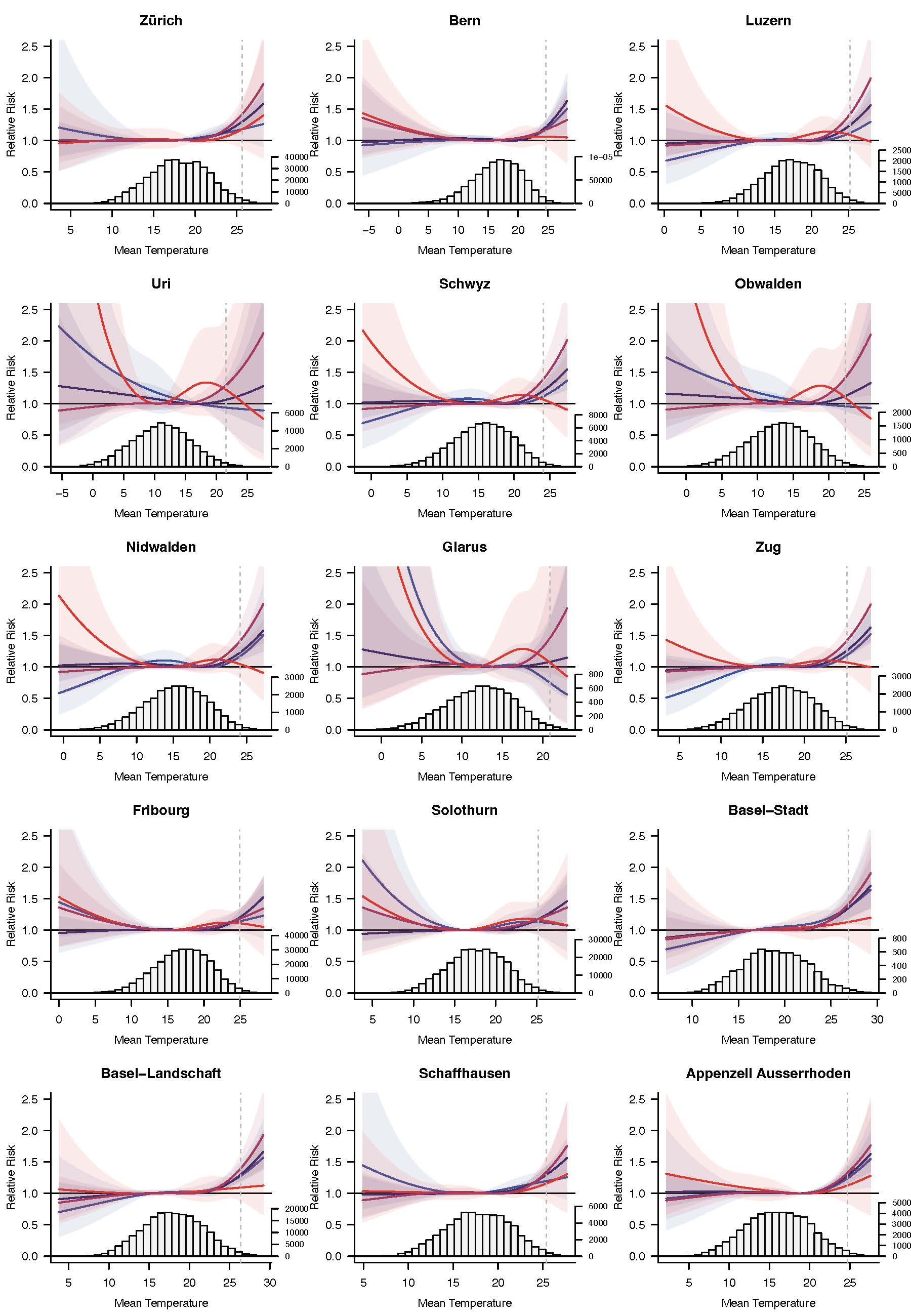


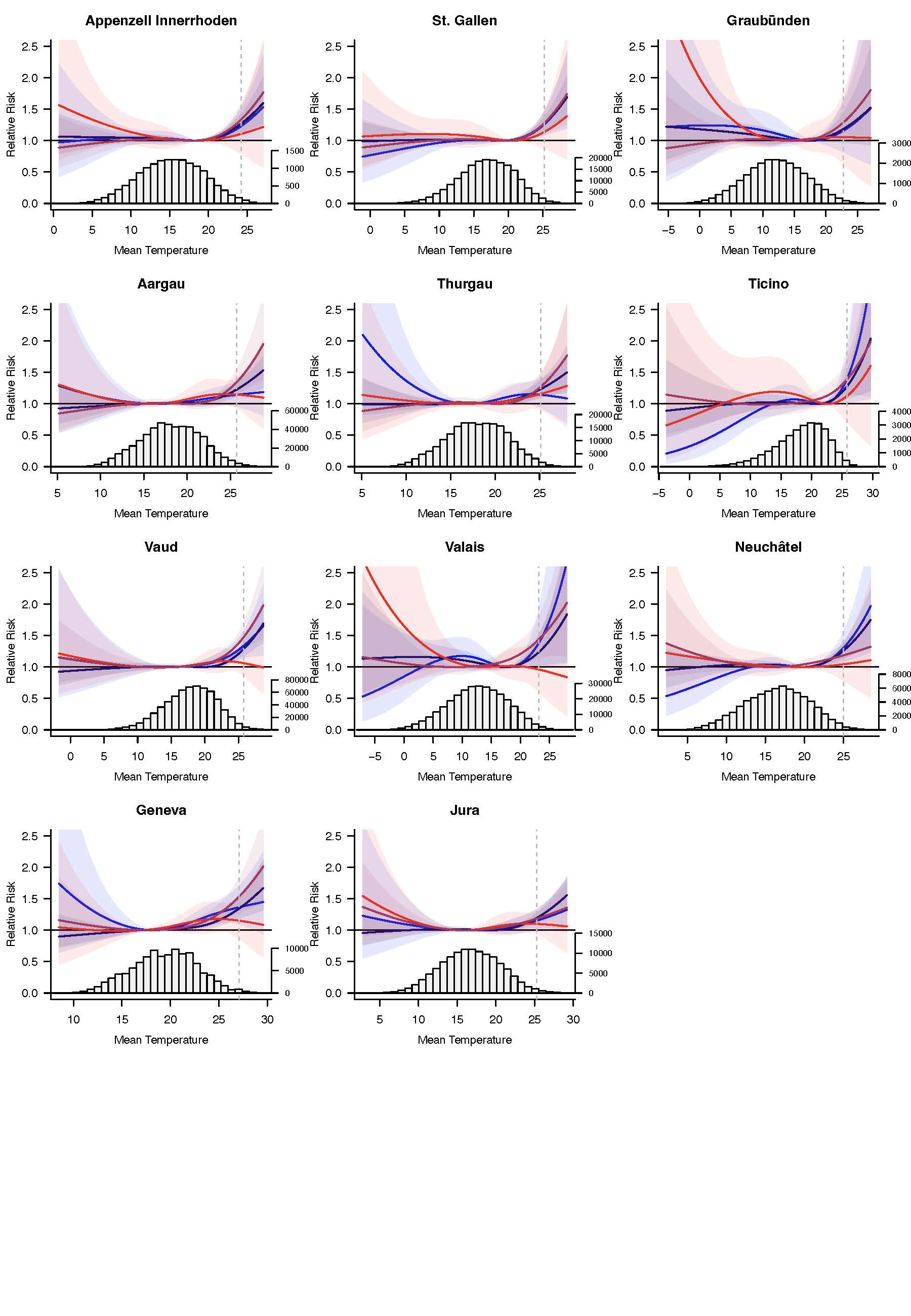


**Suppl. Figure 1.** Temperature-related mortality by age-sex subgroup in the 26 cantons in Switzerland between June-August 1990-2017. The association is expressed in terms of relative risk estimated for each temperature value, using the temperature of minimum mortality as reference. Shaded areas represent the 95% confidence intervals. Light blue: male 0-65 years, dark blue: male >65 years, light red: female 0-65 years, dark red: female >65 years. The plots also show the temperature distribution in each Canton.


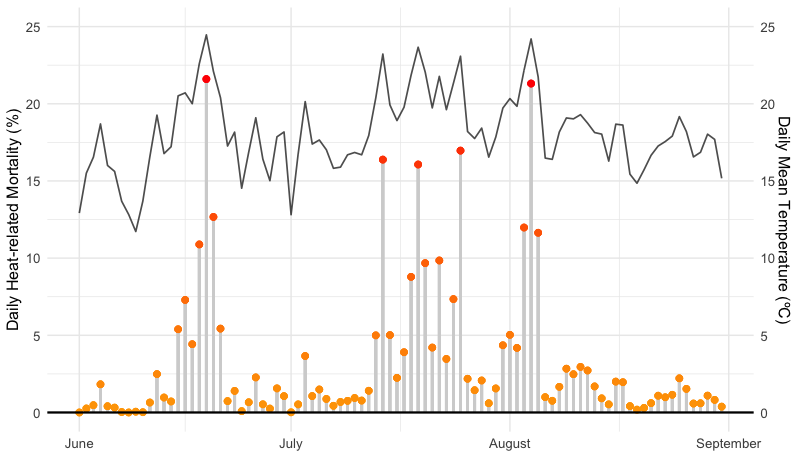


**Suppl. Figure 2.** Daily mean temperature and heat-related mortality (%) in Switzerland between June-August 2022. Heat-related mortality expressed as fraction (%) of all-cause deaths that can be attributed to heat each day.


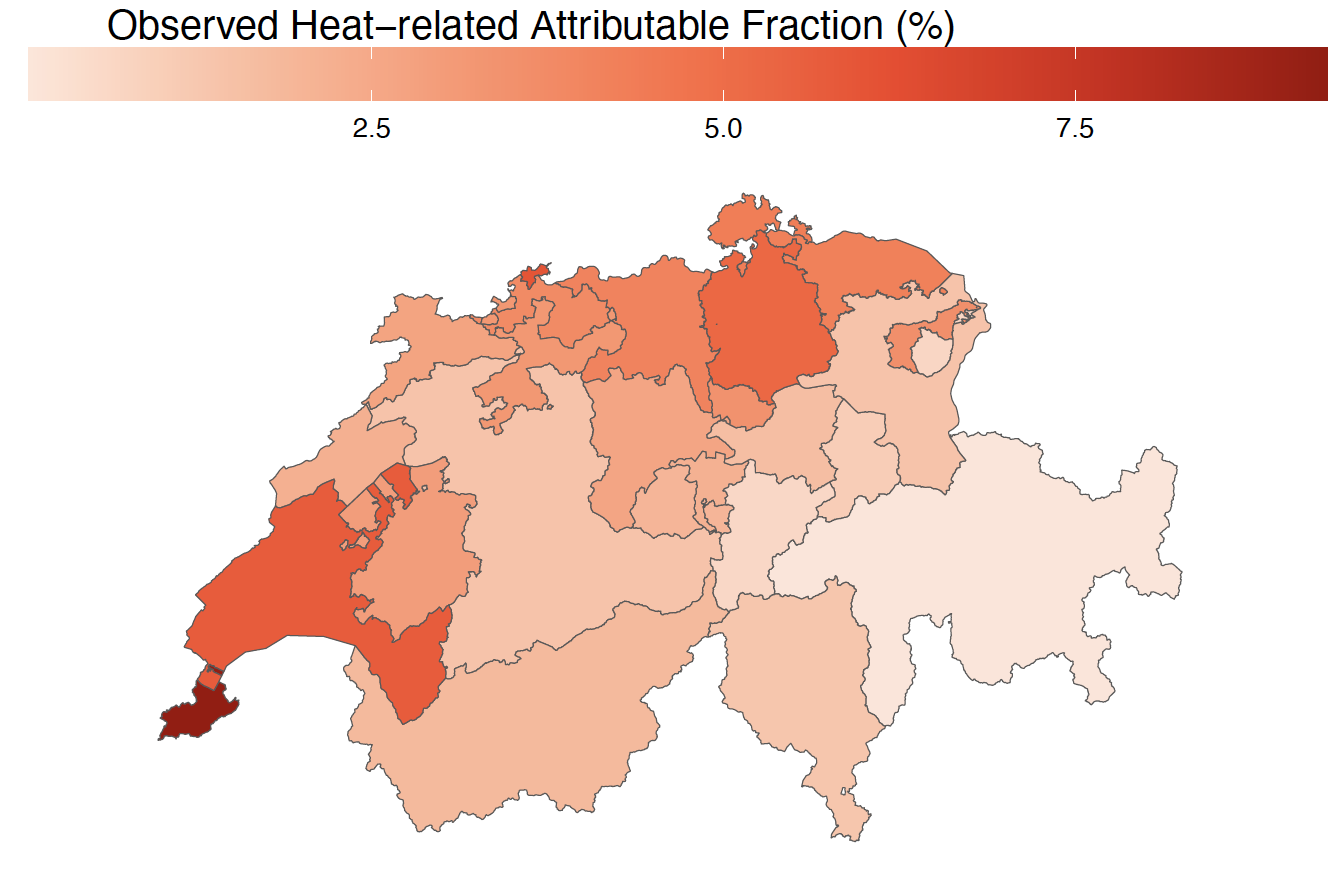

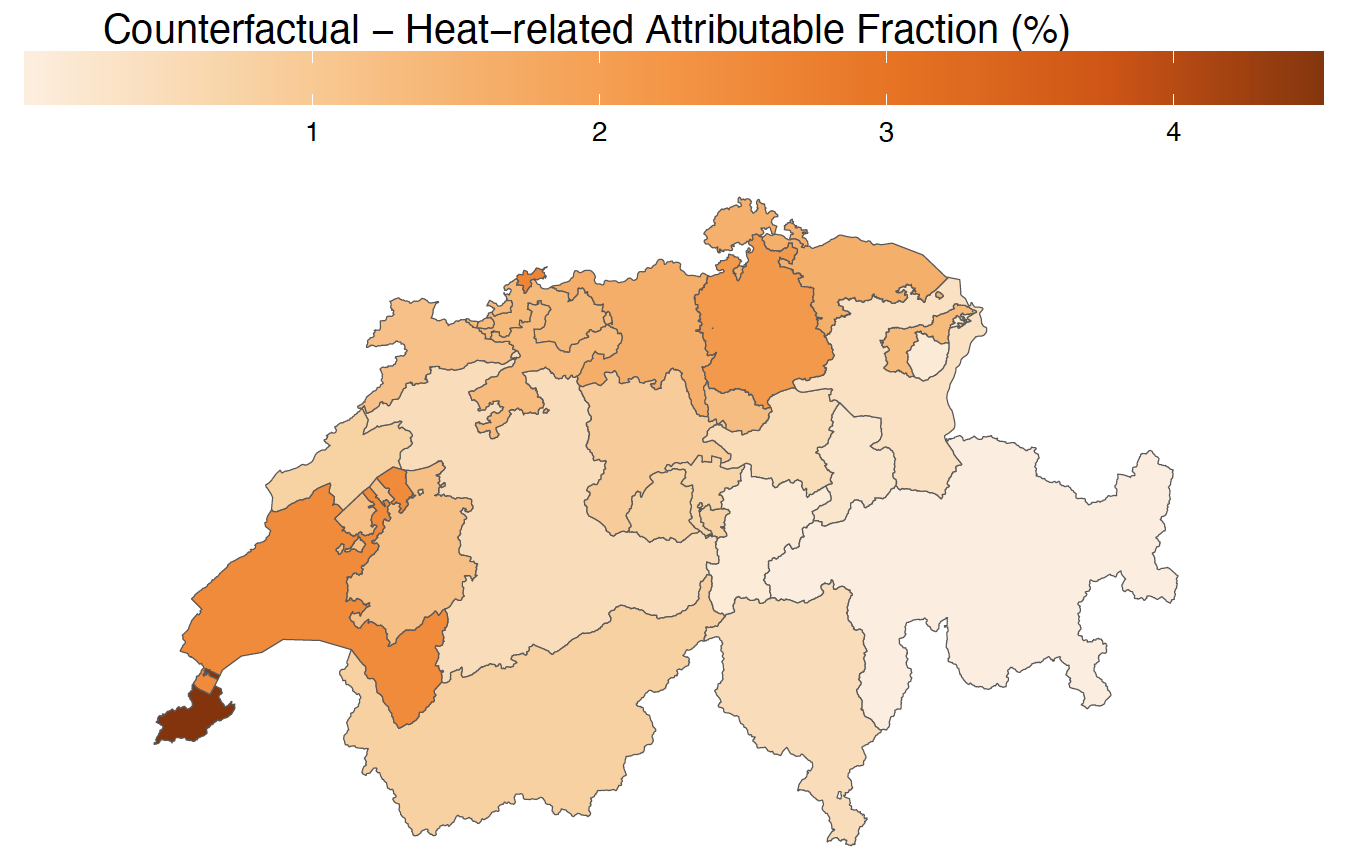


**Suppl. Figure 3.** Canton-specific heat-related mortality fraction (%) in the factual ("observed") and the counterfactual scenario.

**
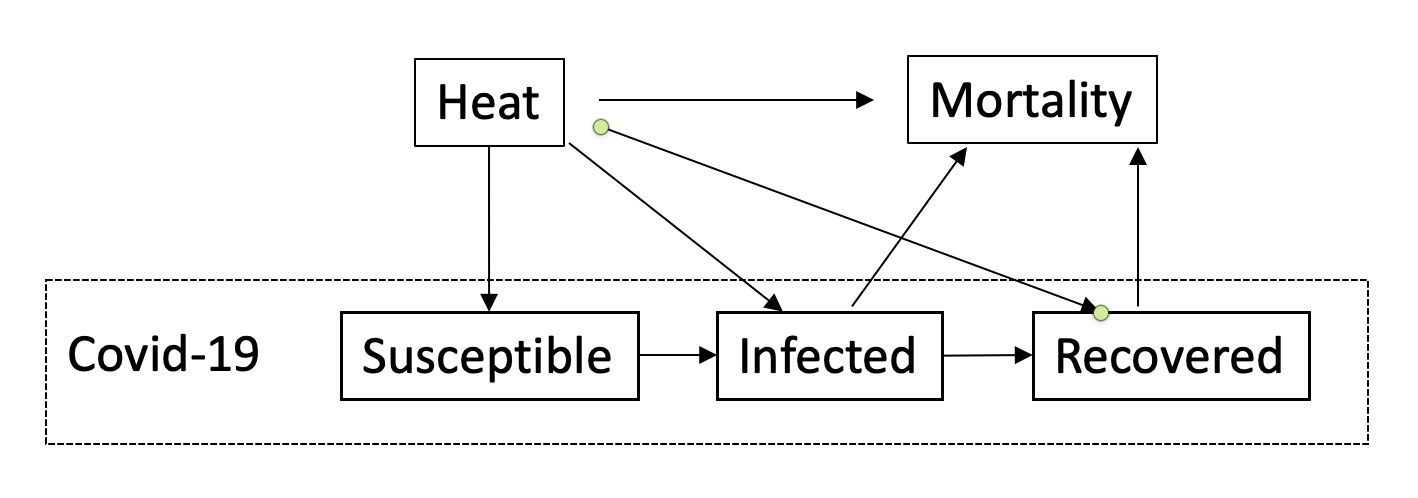
**

**Suppl. Figure 4.** Direct acyclic graph (DAG) depicting the relationship between heat, mortality, and COVID-19.
